# Social media: friend or foe in the COVID-19 pandemic?

**DOI:** 10.6061/clinics/2020/e1953

**Published:** 2020-05-11

**Authors:** Diego Laurentino Lima, Maria Antonieta Albanez A de Medeiros Lopes, Ana Maria Brito

**Affiliations:** I Department of Surgery, Montefiore Medical Center, The Bronx, New York, United States.; II Cardiologia, Real Hospital Portugues, Recife, PE, BR; III Departamento de Saude Coletiva, Instituto Aggeu Magalhaes, Fundacao Oswaldo Cruz; IV Faculdade de Ciencias Medicas da Universidade de Pernambuco, Recife, PE, BR

During the 1918-19 “Spanish” influenza pandemic, people did not have the same sources of communication we now have in the 21^st^ century to quickly share news and information. Social historians have argued that the reason the 1918–19 pandemic left so few traces in public memory is that it was overshadowed by the First World War; hence its historiographical characterization as the ”forgotten pandemic” due to the crucial role played by wartime propaganda 1. For context, a public health report on response to that pandemic in the city of Minneapolis showed that critical information about the virus was spread primarily via postal workers, Boy Scouts, and teachers 2.

With the advent of social media in the 21^st^ century, we are not only learning the latest news but also using platforms like Facebook and Twitter to provide personal and business updates. For businesses, this means leveraging social media to support employees and customers like never before. For the government, it means doing its best to efficiently share factual and up-to-date information 3.

Delivering fast and reliable information is crucial to decrease the transmission of highly contagious infections, not only for healthcare workers but also for the general population 4. The biggest challenge may be to deliver information to the people who are at the battlefront in severely affected areas faster than the dissemination rate of the disease. Many scientific journals have allowed open access for most manuscripts on COVID-19. For health professionals, this may be adequate. However, for the general population, this has no impact on raising awareness. These days, people are overwhelmed by the information they receive on their smartphones through channels such as Facebook, Twitter, WhatsApp, YouTube and Instagram. The biggest problem is in determining which news to trust. Even a pandemic can be used as a political battle, where some will recommend social isolation while others recommend doing nothing that will stop the economy. Who is right, the ones who recommend chloroquine or those who tell you to take your antipyretic medicine and stay home if you have mild symptoms? It is not uncommon to see hundreds of daily texts, videos and even scientific publications in social media groups defending each argument.

We are living not just in a pandemic, but also in an “infodemic” where fake news is becoming more common. These messages and texts always start the same way: they feature a physician, nurse, surgeon, or other authority figure who shares advice—such as holding your breath as a COVID-19 confirmation test, or taking vitamins to decrease the possibility of infection 5. It is understandable that we all want to protect our families and friends and that the lack of answers regarding this new disease increases the level of anxiety in society. It seems as though evidence of the highest level is not as important as social media experts' texts that are broadly shared on the Internet.

Fake news also leads to racism and xenophobia toward Chinese people 6. In Japan, discrimination against Chinese nationals has become widespread: visitors from China have been called bioterrorists, dirty, and insensitive 6. Fake news has led desperate Japanese people to besiege pharmacies to buy surgical masks 6. In Brazil, a similar phenomenon took place with chloroquine, even though scientific studies showed no clear benefits of the use of the drug to treat the COVID-19 infection 7. It is almost impossible to log in to your social media accounts and not see a suspect text or message on any of these topics.

Governments and healthcare authorities should use social media to spread updates, news, and scientific discoveries about COVID-19. Information to be spread can include who should be tested, when they should be tested, and where they would go to get medical care 8. Social media platforms can also provide helpful direction during this crisis. Facebook, for example, redirects users to World Health Organization (WHO) websites where trustworthy information can be obtained 8. Furthermore, social media can be used for education. Physicians are hosting webinars not only to conduct discussions about COVID-19 but also to talk about their specialties, since most conferences have either been canceled or postponed. Another interesting use of social media during this pandemic is to gather data from people who are in lockdown. Information on symptoms, interactions, and travel routes can all be used to fight the virus and better understand how it spreads.

Due to the overflow of information, practical steps should be taken when dealing with the social media infodemic. Information from reliable sources such as government healthcare authorities and specialists should be trusted. Unreliable information should not be circulated before evaluating the sources and their conflicts of interest. Messages with political content that can influence how we deal with this disease should be avoided. It should be kept in mind that this disease has no race, gender, or political affiliation.

Social media is extremely important to fight this contagious disease, not only to get information and be updated about it but also to understand how it spreads, how people interact, and how we can respond to it. As Heidi Tworek, assistant professor at University of British Columbia in Canada, said on Twitter, “Communications in a public health crisis are as crucial as medical intervention … in fact, communications policies ARE a medical intervention” 9. We didn't have this tool a hundred years ago, but now we must use it wisely in every way we can to overcome this pandemic.
